# NFATc3-dependent expression of miR-153-3p promotes mitochondrial fragmentation in cardiac hypertrophy by impairing mitofusin-1 expression

**DOI:** 10.7150/thno.37181

**Published:** 2020-01-01

**Authors:** Tao Wang, Mei Zhai, Sheng Xu, Murugavel Ponnusamy, Yan Huang, Cui-Yun Liu, Man Wang, Chan Shan, Pei-Pei Shan, Xiang-Qian Gao, Kai Wang, Xin-Zhe Chen, Jing Liu, Jing-Yi Xie, De-Yu Zhang, Lu-yu Zhou, Kun Wang

**Affiliations:** 1 Center for Developmental Cardiology, Institute for Translational Medicine, College of Medicine, Qingdao University, Qingdao 266021, China; 2 State Key Laboratory of Cardiovascular Disease, Heart Failure Center, Fuwai Hospital, National Center for Cardiovascular Diseases, Chinese Academy of Medical Sciences, Peking Union Medical College, Beijing, 100037, China

**Keywords:** miR-153-3p, mitofusion-1, NFATc3, mitochondrial fragmentation, cardiac hypertrophy

## Abstract

Mitochondrial dysfunction is involved in the pathogenesis of various cardiovascular disorders. Although mitochondrial dynamics, including changes in mitochondrial fission and fusion, have been implicated in the development of cardiac hypertrophy, the underlying molecular mechanisms remain mostly unknown. Here, we show that NFATc3, miR-153-3p, and mitofusion-1 (Mfn1) constitute a signaling axis that mediates mitochondrial fragmentation and cardiomyocyte hypertrophy.

**Methods:** Isoprenaline (ISO) was used to stimulate the hypertrophic response and mitochondrial fragmentation in cultured cardiomyocytes and *in vivo*. We performed immunoblotting, immunofluorescence, and quantitative real-time PCR to validate the function of Mfn1 in cardiomyocyte hypertrophy. Bioinformatic analyses, a luciferase reporter assay, and gain- and loss-of-function studies were used to demonstrate the biological function of miR-153-3p, which regulates mitochondrial fragmentation and hypertrophy by targeting Mfn1. Moreover, ChIP-qPCR and a luciferase reporter assay were performed to identify transcription factor NFATc3 as an upstream regulator to control the expression of miR-153-3p.

**Results:** Our results show that ISO promoted mitochondrial fission and enhanced the expression of miR-153-3p in cardiomyocytes. Knockdown of miR-153-3p attenuated ISO-induced mitochondrial fission and hypertrophy in cultured primary cardiomyocytes. miR-153-3p suppression inhibited mitochondrial fragmentation in ISO-induced cardiac hypertrophy in a mouse model. We identified direct targeting of Mfn1, a key protein of the mitochondrial fusion process, by miR-153-3p. Also, miR-153-3p promoted ISO-induced mitochondrial fission by suppressing the translation of Mfn1. We further found that NFATc3 activated miR-153-3p expression. Knockdown of NFATc3 inhibited miR-153-3p expression and blocked mitochondrial fission and hypertrophic response in cardiomyocytes.

**Conclusions:** Our data revealed a novel signaling pathway, involving NFATc3, miR-153-3p, and Mfn1, which could be a therapeutic target for the prevention and treatment of cardiac hypertrophy.

## Introduction

Myocardial hypertrophy is a compensatory response of cardiac muscle to a continuously increasing heart load that is mainly characterized by increased myocardial cell volume/surface area, increased cardiac weight, stromal cell proliferation, and myocardial remodeling 1, 2. Pathological cardiac hypertrophy is associated with ventricular remodeling and cardiac dysfunction, which is caused by many cardiovascular diseases 3. Cardiac hypertrophy involves not only in the abnormal increase of myocardial tissue and ventricular wall/interventricular septum 4 but also accompanied by up-regulation of embryonic genes, myocardial fibrosis and cardiac dysfunction 5. Mitochondrial dysfunction is an important contributor to the development of various cardiovascular diseases by enhancing the production of reactive oxygen species and mitochondrial oxidative stress damage, excessive consumption of ATP, and impaired mitochondrial dynamics 6-9. The disequilibrium of mitochondrial dynamics involving aberrant mitochondrial fission and fusion is a contributor to the pathogenesis of cardiac hypertrophy 10-12. However, the molecular link between abnormal mitochondrial dynamics and cardiac hypertrophy is still mostly unknown.

MicroRNAs (miRNAs) are endogenous single-stranded short non-coding RNAs that inhibit protein expression by mediating degradation of mRNA 13. MiRNAs play an important role in the regulation of gene expression at transcriptional and post-transcriptional levels. It is well documented that miRNAs are significant players in key cellular events such as cell proliferation, apoptosis, and development 14, 15. Emerging evidence indicates that miRNAs are critical regulators of the pathogenesis of cardiac hypertrophy. For example, miR-378 inhibits cardiac hypertrophy by suppressing myocardial fibrosis through a paracrine mechanism 16. miR-99a blocks physiological cardiac hypertrophy by downregulating the mTOR signaling pathway 17. In contrast, microRNA-146a promotes the development of cardiac hypertrophy and ventricular dysfunction by reducing dihydrolipoyl succinyltransferase (DLST), which is an important component of oxidative metabolism 18. miR-17-3p contributes to exercise-induced cardiac growth by targeting TIMP-3 and PTEN 19. Although the functions of miRNAs have been extensively studied, there are only a few studies available to demonstrate the effects of miRNAs on mitochondrial dynamics during pathological hypertrophy.

Mitochondrial dynamics refers to the processes of mitochondrial fission and fusion. Aberrant mitochondrial dynamics has been implicated in cardiovascular diseases. The dynamic balance of mitochondrial fission and fusion is tightly regulated by a network of mitochondrial membrane proteins, such as mitofusin 1/2 (Mfn1/2), dynamin-related protein 1 (Drp1), mitochondrial fission factor (MFF) and optic atrophy 1 (OPA1). In mammalian cells, Mfn1, an outer mitochondrial membrane protein, plays a key role in fusion processes of mitochondrial membrane and controls the changes in mitochondrial morphology 20. In cardiac tissue, Mfn1 inhibits apoptosis of cardiomyocytes by attenuating mitochondrial fission and reduces myocardial infarct size in mice with cardiac ischemia/reperfusion injury 21. In recent studies, downregulation of Mfn1 has been observed in cardiac hypertrophy animal models 22, 23. However, the influence of Mfn1 on mitochondrial dynamics during cardiac hypertrophy is not yet fully understood.

The isoform of nuclear factor of activated T cells (NFAT), NFATc3(also known as NFAT4), is generated by NFATc3 gene located on mouse chromosome 8 24. NFATc3 is abundantly expressed in the cardiac muscle and it is a downstream target of calcineurin 25. In cardiomyocytes, NFATc3 promotes mitochondrial fission processes, altered mitochondrial morphology, and cell apoptosis during myocardial infarction 26. Furthermore, NFATc3-dependent pathway has been implicated in the development of cardiac hypertrophy 27, 28. However, the involvement of NFATc3-dependent signaling in the regulation of mitochondrial dynamics during cardiac hypertrophy remains unknown.

Our current study revealed that miR-153-3p is involved in the regulation of mitochondrial networks during the hypertrophic response in cardiomyocytes. We identified that miR-153-3p inhibits translation of Mfn1 and thereby accelerates mitochondrial fission and cardiomyocyte hypertrophy. We also showed that silencing of miR-153-3p inhibits mitochondrial fission and hypertrophic response in cardiomyocytes as well as isoproterenol (ISO)-induced cardiac hypertrophy in mice. We further found that NFATc3 is an inducer of miR-153-3p expression during hypertrophy in cardiomyocytes. Thus, our data uncovered a novel signaling axis and provided new candidates for treating pathological cardiac hypertrophy.

## Materials and Methods

### Primary Cardiomyocytes culture and treatment

Primary cardiac myocytes were isolated from 1-2-day-old mice. Briefly, neonatal mice were cleaned twice with 75% alcohol, and hearts were excised under sterile condition. The dissected hearts were washed in phosphate-buffered saline (PBS) by rinsing 3-4 times, minced, and cardiac cells were enzymatically dispersed using PBS containing 30 mg/mL pancreatin and 10 mg/mL collagenase. The cell suspension was then centrifuged at 1000 rpm for 5min, and cells were resuspended in DMEM/F12 culture medium with 10% fetal bovine serum and plated for 1-2 h. Subsequently, the floating cells (mostly cardiomyocytes) were plated in culture dishes of different sizes according to the experimental requirements. For the elimination of fibroblasts, BrdU (100 μM) was added to the culture medium during the initial 48 h. The cells were kept in a thermostatic incubator and maintained in a humidified atmosphere consisting of 5% CO2 and 95% air. Cardiomyocytes were exposed to isoproterenol (ISO, 10 μM) for the indicated time to induce hypertrophy.

### Adenoviral constructions and infection

Mouse Mfn1 was synthesized using mouse cDNA as the template by standard PCR procedures. Adeno-X expression system (Clontech, Otsu, Japan) was used to construct an adenovirus carrying Mfn1. The mouse Mfn1 RNA interference (siRNA) sequence was 5'-ATACAGGGCTACAGAAACA-3' and the scramble control was 5'-CACTAGATAGCAGAAGAAC-3'. The NFATc3 RNA interference target sequence was 5'-CCCTTTGAGTGCCCAAGTA-3' and control was 5'- ATTCGCCTAGCTGACTGAC-3'. The construction of adenovirus carrying siRNA or its scramble control was carried out according to the manufacturer's instructions using pSilencer adeno 1.0-CMV System (Ambion, Grand Island, NY, USA).

### Transfection of miR-153-3p mimics and antagomirs

MiR-153-3p mimic and negative control mimic were obtained from GenePharma Co. Ltd. (Shanghai, China). The mir-153-3p mimic sequence was 5'-UUGCAUAGUCACAAAAGUGAUC-3'. The miR-153-3p antagomir sequence was 5'-GAUCACUUUUGUGACUAUGCAA-3'. The antagomir-NC sequence was 5'-CAGUACUUUUGUGUAGUACAA-3'. Lipofectamine 3000 (Invitrogen, Grand Island, NY, USA) was used to transfect mimics or antagomirs in cardiomyocytes and transfection was carried out according to the manufacturer's instructions.

### Quantitative reverse real-time PCR

The levels of miR-153-3p and other mRNAs were determined using SYBR green and CFX96 real-time PCR detection system (Bio-Rad). Total RNA was extracted using Trizol reagent (Invitrogen) and RNA was reverse transcribed using reverse transcriptase obtained from ReverTra Ace, (Toyobo). The level of miR-153-3p was normalized to U6. U6 primers were, forward: 5'-GCTTCGGCAGCACATATACTAA-3'; reverse: 5'-AACGCTTCACGAATTTGCGT-3'. Mfn1 forward primer: 5'-GTTTTAGTAGACAGCCCAGG-3'; Mfn1 reverse primer: 5'-GAAGCAGAAGCATCCCAACG-3'. Myocardial hypertrophy induction was determined by the expression levels of ANP and β-MHC genes with the forward primer of ANP: 5'-CTCCGATAGATCTGCCCTCTTGAA-3'; reverse primer: 5'-GGTACCGGAAGCTGTTGCAGCCTA-3'; β-MHC forward primer: 5'- CAGACATAGAGACCTACCTTC-3'; reverse primer: 5'- CAGCATGTCTAGAAGCTCAGG-3'. The expression levels were normalized to glyceraldehyde-3-phosphate dehydrogenase (GAPDH), which was used as an internal control. GAPDH forward primer: 5'-TGTGTCCGTCGTGGATCTGA-3'; reverse: 5'-CCTGCTTCACCACCTTCTTGA-3'.

### Western blot analysis

After various treatments, cardiomyocytes were lysed by using the lysis buffer (150 mM NaCl, 1% Triton X-100, 1% sodium deoxycholate, 0.1% SDS) containing a mixture of protease inhibitors and keeping them on ice for 30min. Equivalent amounts of proteins were loaded onto SDS-PAGE gel and electrophoresis was carried out for 1.5 h at 150 V. Subsequently, the proteins were transferred from the gel to the PVDF membrane and the membrane was blocked with 5% skimmed milk powder in 1% TBST at room temperature for 1 h. The membranes were probed using primary antibodies, including the anti-Mfn1 (1:500), anti-GAPDH (1:5000) and anti-NFATc3 (1:2000) overnight at 4^o^C. Next, the membranes were washed 3 times with 1% TBST and incubated with secondary antibody prepared in 5% milk in 1% TBST for 1 h and then washed with 1%TBST. The blot images were captured by darkroom development using chemiluminescence method.

### Mitochondrial staining

We used MitoTracker Red CMXRos to stain the mitochondria of living cells. The cells were seeded onto the coverslips coated with 0.01% poly-L-lysine for 24 h. After various treatments, the cells were incubated with preheated MitoTracker Red CMXRos staining fluid (0.02 μM) for 25 min under normal conditions. The images of mitochondria were acquired using a laser scanning confocal microscope (Leica TCS SP8). The percentage of mitochondria-disrupted cells relative to the total number of cells was expressed as mean ± S.E.M. At least three separate experiments were performed and a minimum of 300 cells in each group were counted.

### Determination of the cell surface area

To measure the surface area of cardiomyocytes after various treatments, they were fixed in 3.7% formaldehyde in PBS and dehydrated with acetone for 3 min, and then treated with 0.1% Triton X-100 for 20 min. The cells were stained using fluorescent Phalloidin-TRITC (50 mg/ml) at room temperature for 45 min and visualized by laser confocal microscopy. In each group, 30-40 fields and 150-200 cardiomyocytes per microscopic field were examined.

### Luciferase activity assay

3'UTR of Mfn1 was generated by PCR using the forward primer 5'-CCCTAGATGCACCTCTTATTT-3' and the reverse primer 5'- TTCATCTTGCAAGCTTGCATAC-3'. The PCR amplified 3'UTR of Mfn1 was subcloned into the pGL3 vector (Promega) at a site immediately downstream of the stop codon of the luciferase gene. To construct mutated Mfn1-3'UTR, QuikChange II XL Site-Directed Mutagenesis Kit (Stratagene) was used with the wild type vector of Mfn1-3'UTR as a template. Luciferase activity was measured using the Dual-Luciferase Reporter Assay System (Promega) according to the instructions from the manufacturer. The plasmid constructs (200 ng/well) of wild type 3'UTR or mutated 3'UTR were co-transfected along with miR-153-3p mimic or mimic-NC into HEK-293 cells using Lipofectamine 2000 (Invitrogen, Carlsbad, USA). The luciferase activity was measured at 48 h post-transfection.

### Construction of mouse miR-153-3p promoter

The promoter region of miR-153-3p was amplified from the mouse genome using the forward primer 5'-CCAAAGGCACAGTAAATCGTT-3' and the reverse primer 5'-GTCACACAGAAAATGCTTTCTC-3'. The miR-153-3p promoter fragment was cloned into the vector pGL4.17 (Promega). For the mutations in the putative NFATc3-binding site, the wild type vector was used as a template, and mutants were generated using QuikChange II XL site-directed mutagenesis kit (Stratagene). The constructs were then subjected to sequencing to confirm the presence of desired mutations.

### Chromatin immunoprecipitation (ChIP) assay

Cardiomyocytes were washed with PBS and fixed with 1% formaldehyde at room temperature for 10 min. The cross-linking reaction was quenched by adding glycine (0.1 M) and incubating for 5 min with gentle shaking. Subsequently, the cells were washed twice with cold PBS and cell lysate was prepared using ice-cold cell lysis buffer at 4 °C for 1 h. The cell lysate was sonicated for the fragmentation of chromatin to an average length of 500 to 800 bp. The samples were precleared with Protein-A agarose (Roche) by gentle rotation at 4°C for 1 h. Then specific antibodies were added and kept at 4°C overnight on the rotator. To capture immunoprecipitates, salmon sperm DNA (10% vol/vol) was used to block Protein-A agarose. The purification of captured DNA fragments was carried out using QIAquick Spin Kit (Qiagen). The purified chromatin templates were amplified using qRT-PCR. The following primers were used to detect the binding of NFATc3 to the promoter region of miR-153-3p. Forward primer: 5'- GGCTCAAGTGTGATTCATACT-3' and reverse primer: 5'- GAGTCAATACTCTTAAGGCATC-3'.

### Animal experiments

Adult male C57BL/6 mice (10-wk-old) were infused with ISO (40 mg/kg/day) to induce cardiac hypertrophy, or the same volume of saline, for 14 days using implanted osmotic minipumps (Alzet model 1002, Alza Corp.). For *in vivo* transfer of antagomir or negative control (anta-NC), the mice had infused ISO along with miR-153-3p antagomir (30 mg/kg) or anta-NC (30 mg/kg). The osmotic pumps were surgically removed at the end of the treatment period. All mice were subjected to echocardiographic measurements and hypertrophic analysis after 14 days.

### Histological analysis

At the end of the experimental period, the hearts were excised and immediately fixed in 10% formalin. Subsequently, they were embedded in paraffin using a standard procedure, sectioned into 7 µm slices, and stained with hematoxyline-eosin (H&E). The sections were stained with fluorescein isothiocyanate-conjugated wheat germ agglutinin (Sigma) to measure the cross-sectional area of cardiomyocytes.

### Echocardiographic assessment

Transthoracic echocardiography was performed using a Vevo 2100 imaging system (Visualsonics, Toronto, ON, Canada) with a real-time linear-array scan head (MS-400). Two-dimensional guided M-mode tracing images were obtained in both parasternal long- and short-axis views at the level of papillary muscles. Fractional shortening (FS) was calculated using the standard equation. All measurements were averaged from at least three consecutive beats.

### Statistical analysis

All data were presented as the mean ± SD of at least three independent experiments. We used one-way analysis of variance (ANOVA) followed by Tukey post hoc test for multiple comparisons and p<0.05 was considered statistically significant.

## Results

### ISO induces mitochondrial fission and hypertrophic response in cardiomyocytes

**Figure 1:**
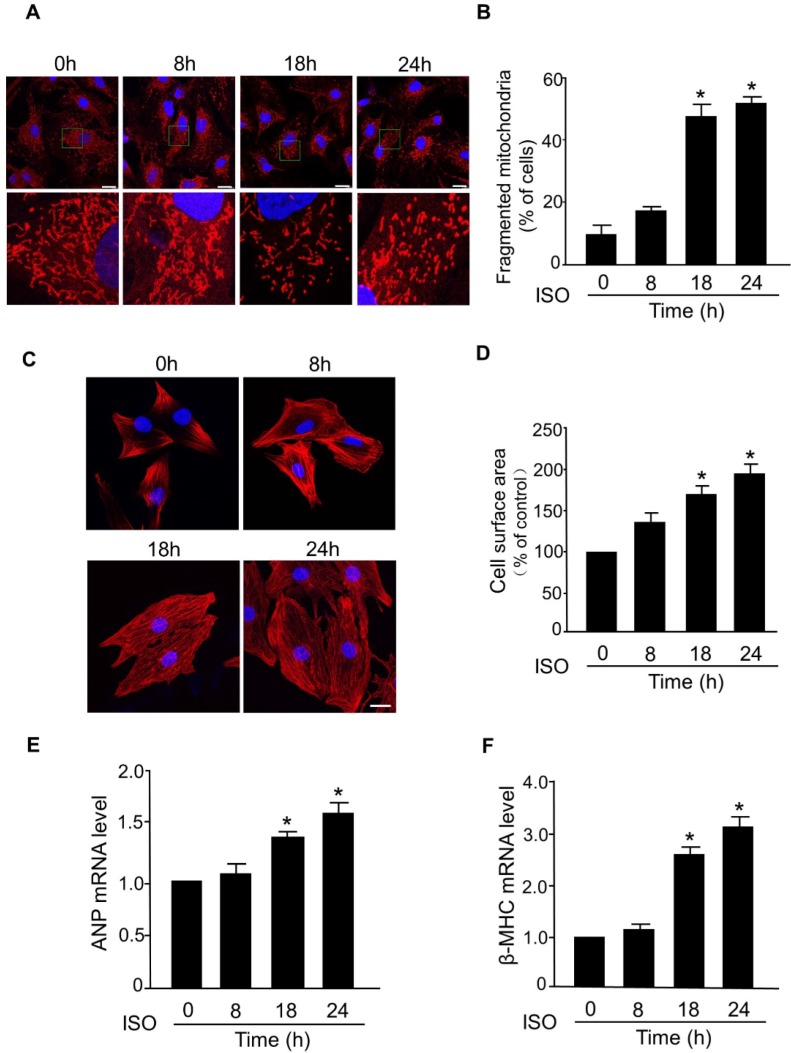
** Mitochondrial fission is involved in ISO-induced cardiomyocyte hypertrophy. (A** and** B)** Cardiomyocytes were treated with ISO (10 μM) for the indicated time and cells were then stained with Mito-Tracker red and DAPI was used to stain nuclei. (A) Representative confocal images of mitochondrial fragmentation in cardiomyocytes. (B) The cardiomyocytes with fragmented mitochondria were counted. Bar = 20 µm.** (C** and** D)** Cardiomyocytes were exposed to 10 μM of ISO for the indicated time and then they were stained with phalloidin-TRITC. (C) Representative images of sarcomere organization in cardiomyocytes. Bar = 10 µm. (D) Quantitative analysis of cell surface area. **(E** and** F)** Cardiomyocytes were treated with ISO (10 μM) and collected at the indicated time. The mRNA levels of ANP (E) and β-MHC (F) were detected by qRT-PCR. *p < 0.05.

The abnormal mitochondrial dynamics is an important contributor to the myocardial cell death and progression of myocardial infarction 29-31. However, the dysregulation of mitochondrial dynamics during the hypertrophic response in cardiomyocytes is still poorly understood. In this study, we investigated whether mitochondrial fission is involved in the development of hypertrophy in cardiomyocytes induced by ISO, a well-known stimulator of hypertrophic response. In cultured primary cardiomyocytes, exposure to ISO significantly increased the level of fragmented mitochondria, an indicator of mitochondrial fission, in a time-dependent manner (Figure 1A-B). ISO-induced hypertrophy was confirmed by a significant increase in the cell surface area (Figure 1C-D) and increased levels of mRNAs of hypertrophy markers (ANP and β-MHC) (Figure 1E-F). Notably, the time-dependent increase of mitochondrial fission was accompanied by a progressive increase of cell surface area and hypertrophy markers in cardiomyocytes exposed to ISO. These results indicated a direct association between aberrant mitochondrial fission and hypertrophic responses in cardiomyocytes.

### Mfn1 regulates ISO-induced mitochondrial fission and hypertrophy in cardiomyocytes

**Figure 2:**
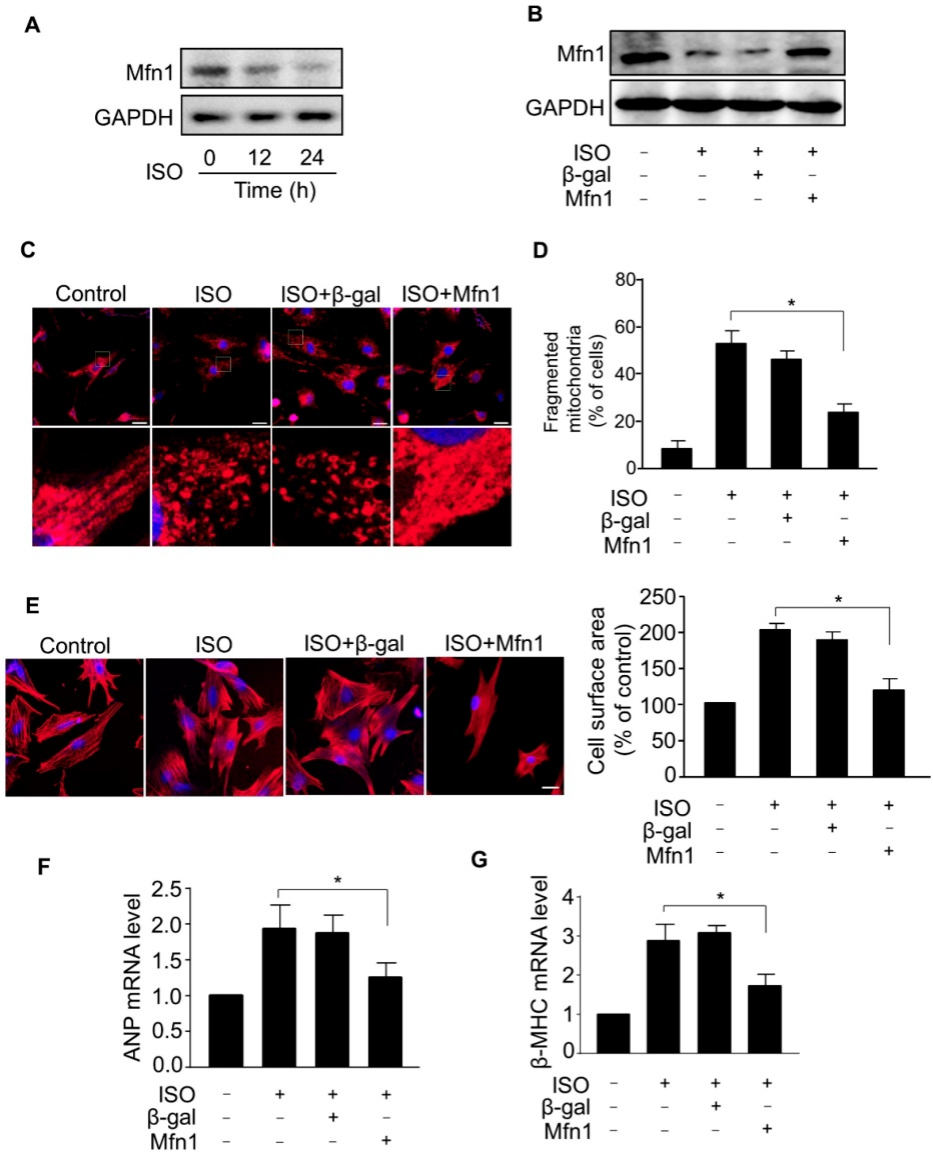
** Mfn1 inhibits mitochondrial fragmentation and hypertrophy in cardiomyocytes. (A)** Cardiomyocytes were exposed to ISO (10 μM) for the indicated time and the level of Mfn1 protein was detected by immunoblot. **(B)** Cardiomyocytes were infected with adenoviruses harboring Mfn1 or β-gal for 24 h, then incubated with ISO for additional 24 h. The expression level of Mfn1 protein was detected by immunoblot. **(C** and** D)** Mfn1 inhibits mitochondrial fission in hypertrophied cardiomyocytes. Cardiomyocytes were treated as described in (B).** (C)** Representative confocal images showing the level of mitochondrial fission in cardiomyocytes. **(D)** Quantitative analysis of the percentage of cells with mitochondrial fragmentation. **(E-G)** Mfn1 inhibits ISO induced hypertrophy in cardiomyocytes. Cardiomyocytes were treated as described in (B). Representative images of sarcomere organization in cardiomyocytes (E, left panel). Bar = 10 µm. Quantitative analysis of cell surface area (E, right panel). The mRNA levels of ANP (F) and β-MHC (G) were detected by qRT-PCR. *p < 0.05.

To understand the mechanism of aberrant mitochondrial fission during ISO-induced hypertrophy, we examined the expression level of Mfn1, a mitochondrial fusion protein, which plays a crucial role in the structural integrity of mitochondria by balancing mitochondrial dynamic networks 32-34, preserving mitochondria from shortening (fragmentation) and preventing the alteration of morphology 21, 23. Our results showed that the level of Mfn1 protein was markedly decreased in cardiomyocytes treated with ISO (Figure 2A), indicating that Mfn1 contributed to the regulation of cardiomyocytes hypertrophy. Next, we explored whether Mfn1 regulated ISO-induced mitochondrial fission in cardiomyocytes. We overexpressed Mfn1 using adenoviral vector and investigated its effect on mitochondrial fission during the hypertrophic response in cardiomyocytes. As shown in Figure 2B, the ISO-induced reduction of Mfn1 level was significantly reversed in cardiomyocytes overexpressing Mfn1. The enhanced expression of Mfn1 significantly inhibited ISO-induced mitochondrial fission in cardiomyocytes as indicated by a reduced level of fragmented mitochondria, (Figure 2C and D). Furthermore, overexpression of Mfn1 significantly decreased ISO-induced increase of cell surface area (Figure 2E) as well as mRNA levels of hypertrophic markers in cardiomyocytes (Figure 2F-G). Collectively, these data suggested that Mfn1 can inhibit mitochondrial fission and hypertrophic response in cardiomyocytes. However, the decline in the level of Mfn1 due to hypertrophy stimulation leads to increased mitochondrial fission process and hypertrophic growth of cardiomyocytes.

### miR-153-3p regulates the expression of Mfn1

**Figure 3:**
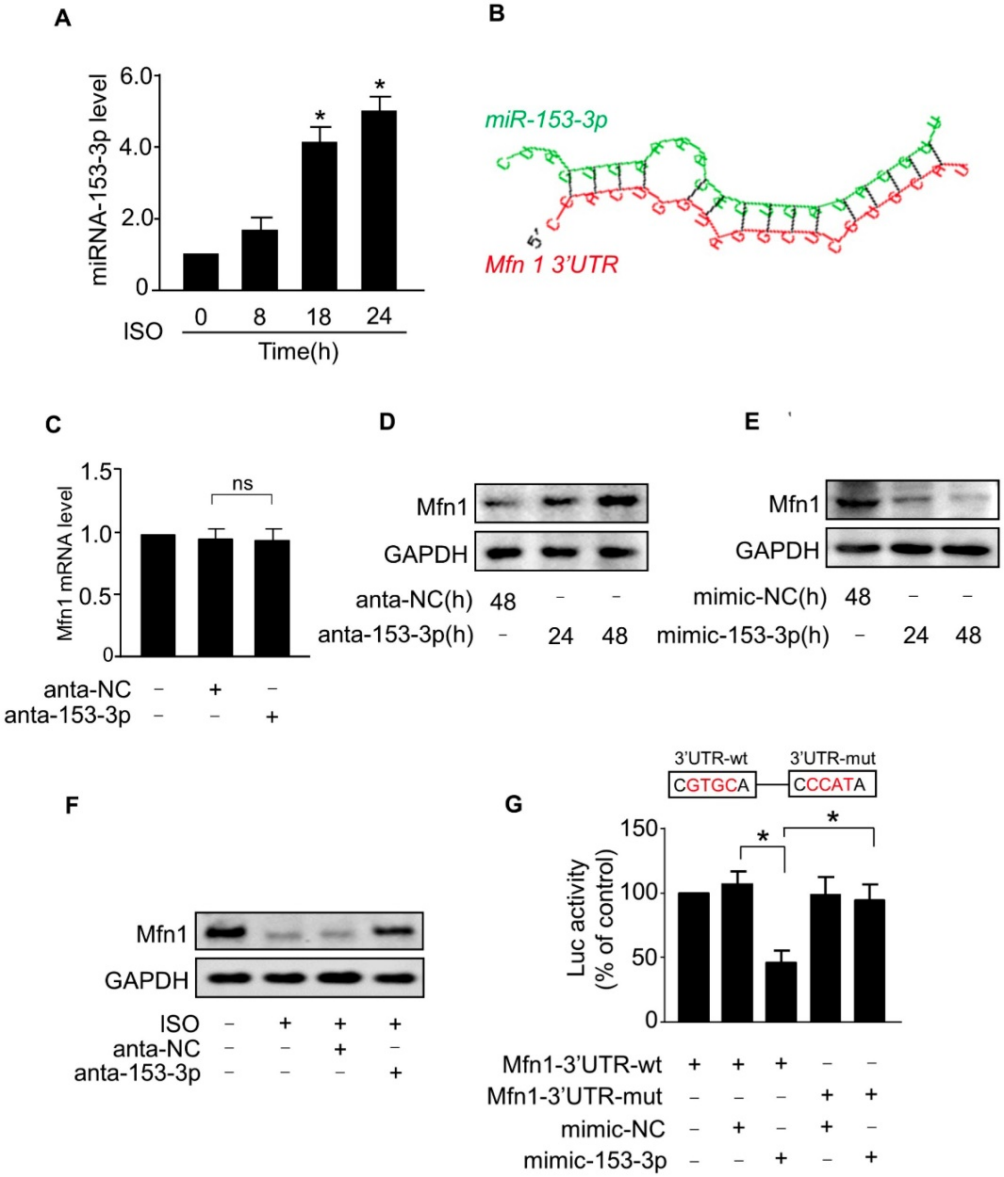
** miR-153-3p regulates Mfn1 expression. (A)** Cardiomyocytes were exposed to ISO and harvested at indicated times. The expression level of miR-153-3p was analyzed by qRT-PCR. *p < 0.05 vs 0 h. **(B)** Analysis of Mfn1 3'UTR region for the potential binding site of miR-153-3p. **(C)** Knockdown of miR-153-3p did not affect the level of Mfn1 mRNA. Cardiomyocytes were transfected with antagomir of miR-153-3p (anta-153-3p) or antagomir-negative control (anta-NC) and the level of Mfn1 mRNA was detected by qRT-PCR. **(D)** Knockdown of miR-153-3p increases Mfn1 protein level. Cardiomyocytes were treated as described in (C). The level of Mfn1 protein was detected by immunoblot. **(E)** Cardiomyocytes were transfected with miR-153-3p mimic (mimic-153-3p) or its negative control (mimic-NC), and Mfn1 protein level was detected by immunoblot.** (F)** Cardiomyocytes transfected with anta-153-3p or anta-NC were exposed to ISO and the level of Mfn1 protein was detected by immunoblot. **(G)** miR-153-3p inhibits translation of Mfn1 mRNA. HEK293 cells were cotransfected with the luciferase construct carrying wild-type Mfn1-3'UTR (Mfn1-3'UTR-wt) or mutated Mfn1-3'UTR (Mfn1-3'UTR-mut) and mimic-153-3p or mimic-NC and cells were harvested for the measurement of luciferase activity. *p < 0.05.

In general, miRNAs negatively regulate gene expression by binding to the 3'-untranslated region (UTR) region of mRNAs. To investigate whether miRNA(s) suppressed the expression of Mfn1 and promoted ISO-induced mitochondrial fission in cardiomyocytes, we first analyzed the 3'UTR region of Mfn1 by the Targetscan and RNAhybrid program and observed that Mfn1-3'UTR contains several potential miRNA binding sites. We then tested the levels of these miRNAs in cardiomyocytes exposed to ISO (data not shown) and found that only miR-153-3p level was remarkably upregulated in cardiomyocytes in response to ISO treatment (Figure 3A). Also, analysis of Mfn1-3'UTR using the RNAhybrid program showed that Mfn1 might be a potential target of miR-153-3p (Figure 3B). We, therefore, selected miR-153-3p for further investigation.

Next, we tested the effect of miR-153-3p on Mfn1 expression. The knockdown of miR-153-3p using antagomir did not alter the level of Mfn1 mRNA (Figure 3C), while miR-153-3p silencing resulted in a significant increase of Mfn1 protein level in cardiomyocytes (Figure 3D). In contrast, enforced expression of miR-153-3p significantly decreased the level of Mfn1 protein in cardiomyocytes (Figure 3E). Also, ISO-induced reduction of Mfn1 protein expression was blocked upon knockdown of miR-153-3p (Figure 3F). To investigate the specificity of regulation of Mfn1 expression by miR-153-3p, we examined the levels of other mitochondrial dynamics-related proteins. The results showed that miR-153-3p knockdown alone or miR-153-3p knockdown along with ISO treatment did not affect the expression of other proteins (Figure S1A-B), indicating specific regulation of Mfn1 by miR-153-3p.

We further examined the influence of miR-153-3p on the translation of Mfn1 using luciferase assay. We constructed wild-type Mfn1-3'UTR vector containing the binding site of miR-153-3p (Mfn1-3'UTR-wt) or its mutant (Mfn1-3'UTR-mut) (Figure 3G, upper panel) and cloned them into a luciferase vector. The luciferase reporter assay showed that miR-153-3p decreased the translation level of wild-type 3'UTR of Mfn1 but did not show any inhibitory effect on the mutated Mfn1-3'UTR (Figure 3G, lower panel). To explore whether the inhibition of miR-153-3p on Mfn1 expression was binding site-dependent, we co-transfected miR-153-3p with wild-type 3'UTR of Mfn1 (Mfn1-3'UTR-wt) or mutated 3'UTR (Mfn1-3'UTR-mut). The results showed that enforced expression of miR-153-3p significantly inhibited the expression of Mfn1-3'UTR-wt (Figure S2A), but exhibited no distinct effect on the expression of Mfn1-3'UTR-mut (Figure S2B), indicating that the effect of miR-153-3p on Mfn1 expression was binding site-dependent. Collectively, these results revealed that Mfn1 is a specific target of miR-153-3p in ISO-induced hypertrophy in cardiomyocytes.

### Knockdown of miR-153-3p suppresses ISO-induced mitochondrial fission and cardiomyocyte hypertrophy

**Figure 4:**
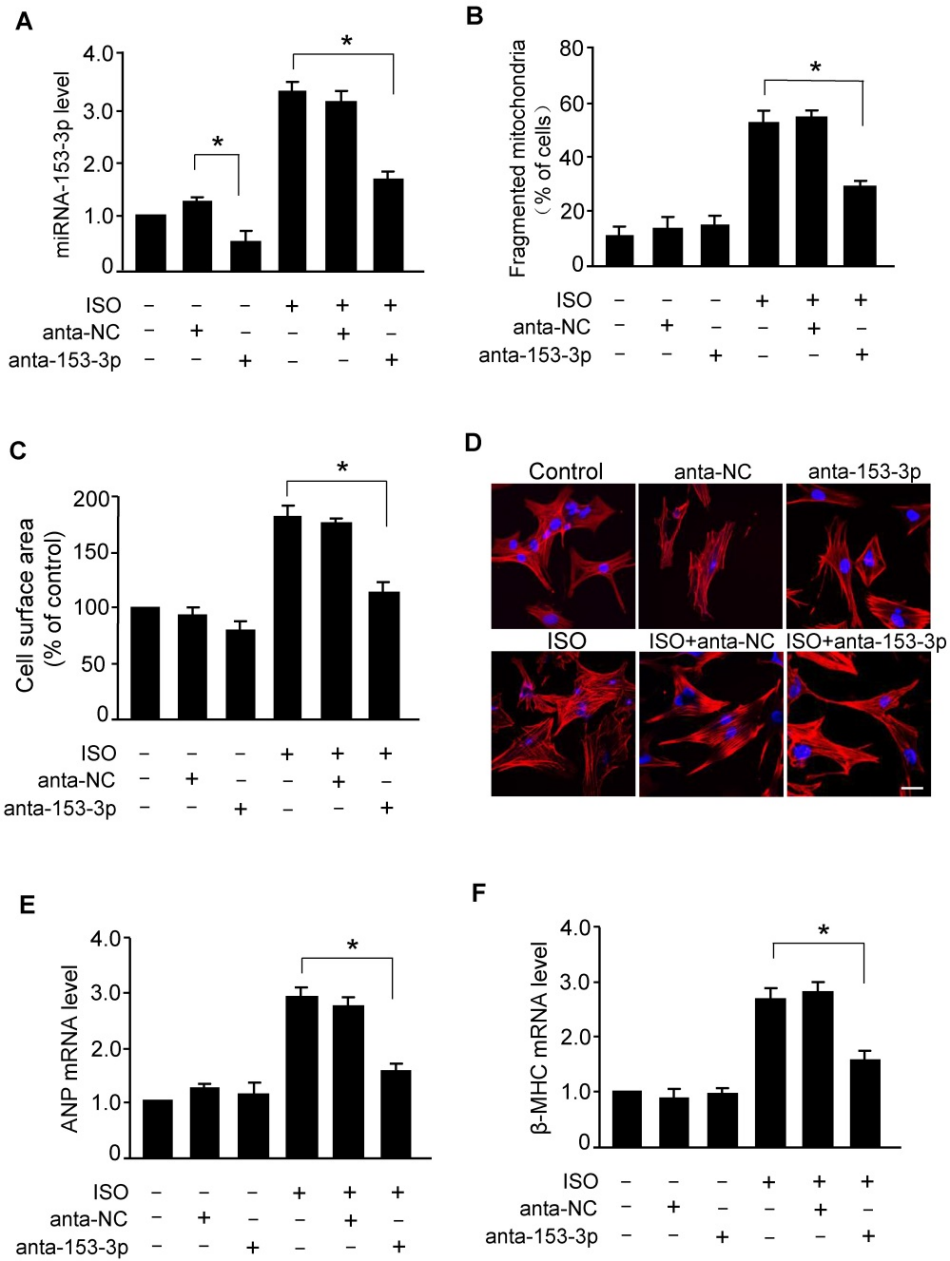
** Knockdown of miR-153-3p suppresses ISO-induced mitochondrial fission and hypertrophy in cardiomyocytes. (A)** Cardiomyocytes were transfected with anta-153-3p or anta-NC and exposed to ISO. The level of miR-153-3p was determined by qRT-PCR. *p < 0.05. **(B)** Knockdown of miR-153-3p inhibits ISO-induced mitochondrial fragmentation. Cardiomyocytes were treated as described in (A). Quantification of the percentage of cardiomyocytes with fragmented mitochondria. **(C**-**F)** Cardiomyocytes were treated as described in (A). Quantitative measurement of cell surface area (C). Representative images of sarcomere organization in cardiomyocytes (D). Bar = 20 µm. The mRNA levels of ANP (E) and β-MHC (F) were detected by qRT-PCR. *p < 0.05.

To examine whether miR-153-3p regulated mitochondrial fission in hypertrophied cardiomyocytes, we treated cardiomyocytes with miR-153-3p antagomir or antagomir-NC and exposed to ISO. As shown in Figure 4A, miR-153-3p antagomir effectively reduced the ISO-induced increase in the level of miR-153-3p. The deficiency of miR-153-3p resulted in attenuation of ISO-induced elevation of mitochondrial fragmentation (Figure 4B) along with a remarkable reduction of ISO-induced increase of cell surface area in cardiomyocytes (Figure 4C and D). The suppression of ISO-induced hypertrophy in miR-153-3p antagomir-treated cardiomyocytes was further confirmed by a significant reduction of ANP (Figure 4E) and *β*-MHC (Figure 4F) levels. These data indicated that miR-153-3p promotes mitochondrial fission and hypertrophy in cardiomyocytes, and inhibition of miR-153-3p can effectively attenuate the hypertrophic response.

### Knockdown of miR-153-3p attenuates mitochondrial fission and cardiac hypertrophy *in vivo*

**Figure 5:**
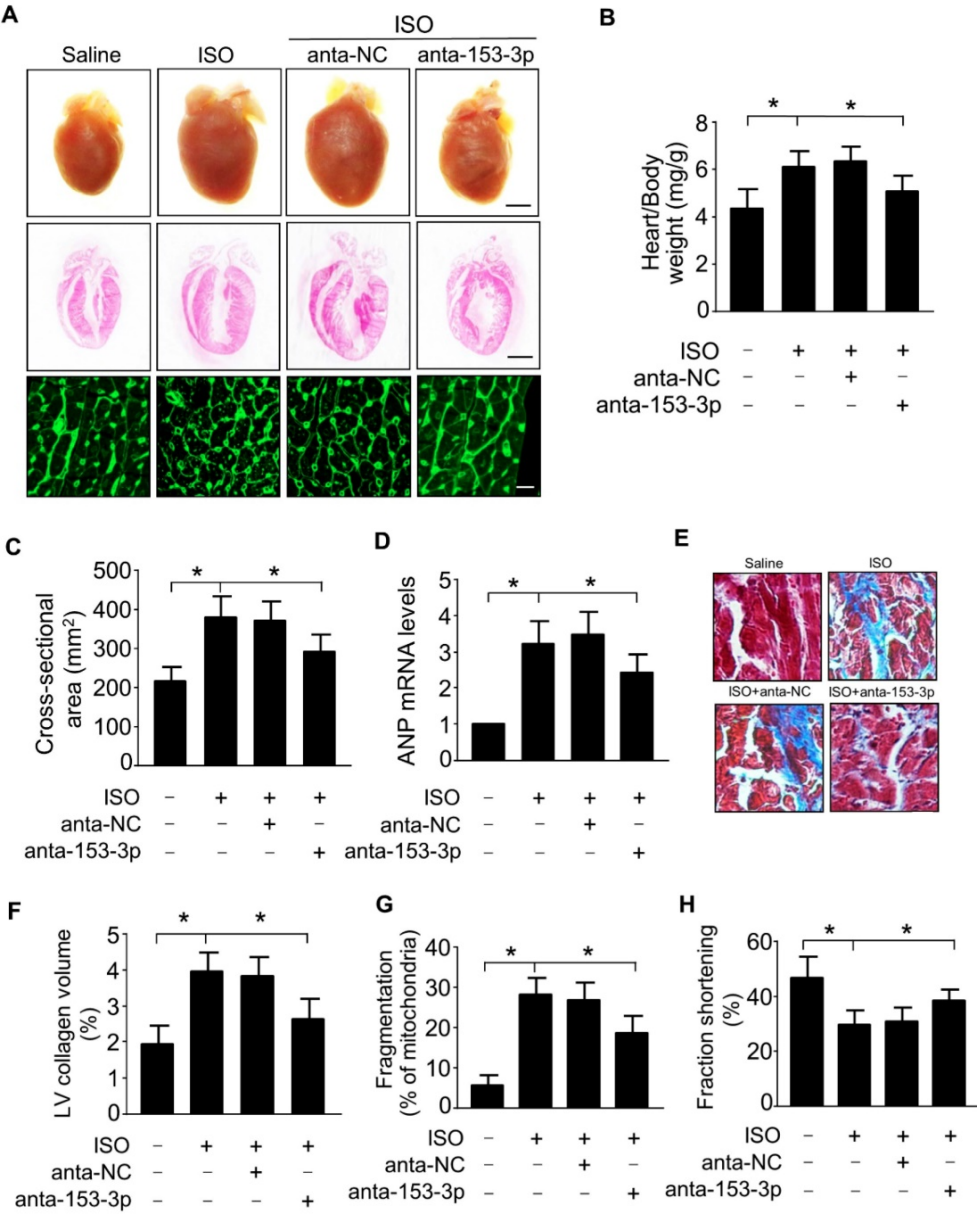
** miR-153-3p regulates cardiac hypertrophy and mitochondrial fission in the heart.** ISO was infused in adult male C57BL/6 mice along with or without anta-153-3p or anta-NC. **(A)** The gross heart morphology (upper panel, Bar = 2 mm), sagittal section of hearts stained with hematoxylin and eosin (middle panel, Bar = 2 mm) and wheat germ agglutinin staining for cell size measurement (lower panel, Bar = 20µm). **(B)** The ratio of heart/body weight. (n=8).** (C)** The cross-sectional area of cardiac myocytes was analyzed by staining with fluorescein isothiocyanate-conjugated wheat germ agglutinin (n=7). **(D)** The expression level of ANP (n=6). **(E** and** F)** Detection of the fibrotic area using Masson's trichrome staining (n=8).** (G)** The percentage of fragmented mitochondria were calculated. n=6. *p < 0.05. **(H)** Echocardiography analysis showing increased fractional shortening in anta-153-3p -treated mice. n=7. *p < 0.05.

To determine the role of miR-153-3p in the development of cardiac hypertrophy, we examined the effects of miR-153-3p antagomir *in vivo* by inducing cardiac hypertrophy in mice. Administration of miR-153-3p antagomir along with ISO attenuated ISO-induced pathological cardiac hypertrophy evident from enlargement of the heart, increased heart to body weight ratio, an increased cross-sectional area of cardiac myocytes, and increased level of ANP mRNA (Figure 5A-D). Also, miR-153-3p antagomir significantly blocked the ISO-induced increase of interstitial fibrotic area and collagen deposition in the hearts of mice (Figure 5E and F). Further, ISO-induced elevation of mitochondrial fission in mouse hearts was remarkably decreased upon administration of miR-153-3p antagomir (Figure 5G). Echocardiography showed that ISO caused cardiac dysfunction indicated by a remarkable reduction of fractional shortening, and miR-153-3p antagomir treatment significantly improved the cardiac function in hypertrophied mouse hearts (Figure 5H). Together, these data suggested that inhibition of miR-153-3p can alleviate pathological changes caused by the hypertrophic response and improve cardiac function by suppressing mitochondrial fission provoked by hypertrophic stimulants such as ISO.

### miR-153-3p targets Mfn1 to promote mitochondrial fission and cardiomyocyte hypertrophy

**Figure 6:**
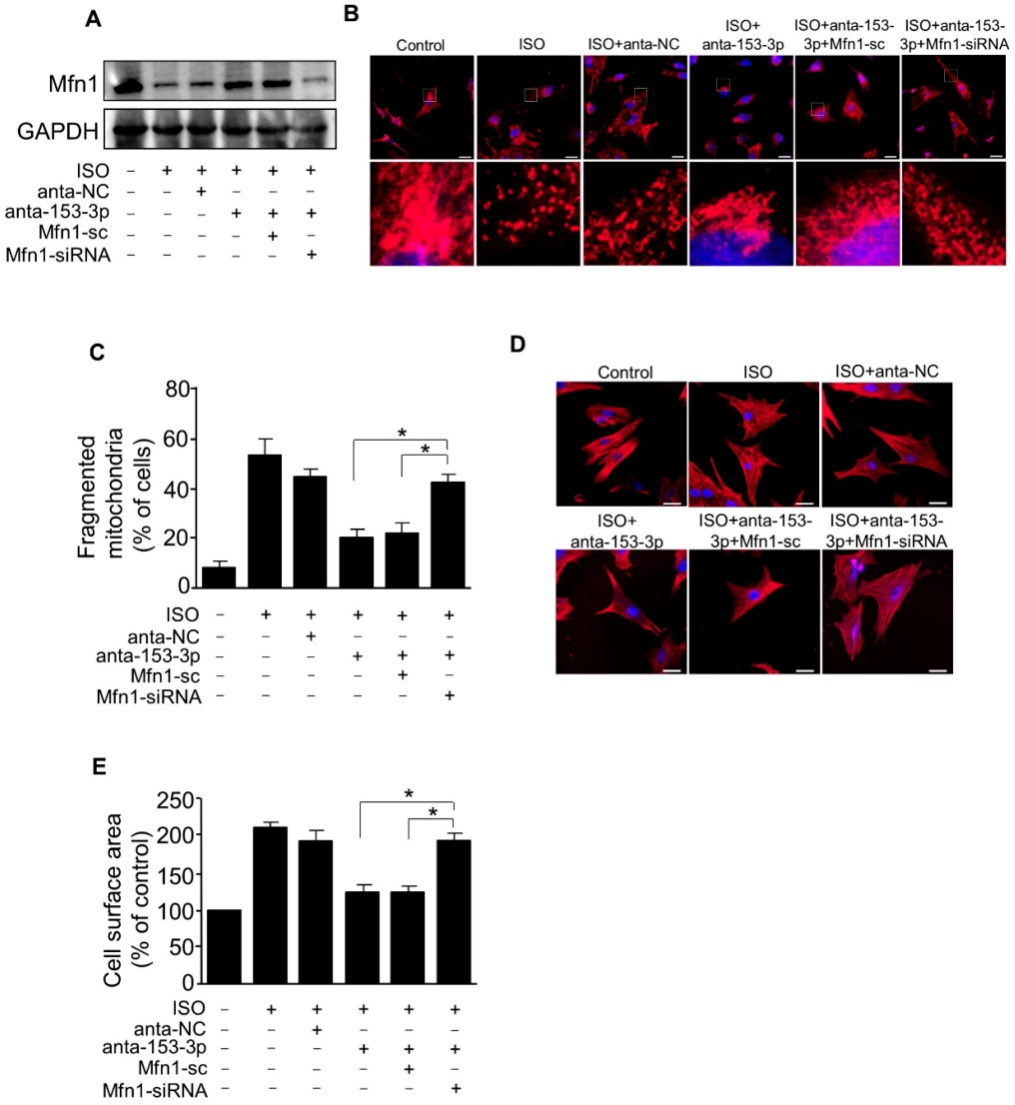
** miR-153-3p increases mitochondrial fission and hypertrophic response by targeting Mfn1.** Cardiomyocytes were simultaneously infected with adenovirus Mfn1-siRNA or its scramble form and transfected with anta-153-3p or anta-NC, and then exposed to ISO. **(A)** The level of Mfn1 protein was detected by immunoblot. **(B)** Representative confocal images showing mitochondrial fission in cardiomyocytes. **(C)** Quantitative measurement of the percentage of cardiomyocytes with fragmented mitochondria. *p < 0.05. **(D)** Representative images of sarcomere organization in cardiomyocytes. Bar = 20 µm. **(E)** Quantitative measurement of the cell surface area. *p < 0.05.

We demonstrated that miR-153-3p and Mfn1 regulated mitochondrial fission and hypertrophy in cardiomyocytes, and miR-153-3p could inhibit the expression of Mfn1 protein. We next verified the relationship between miR-153-3p and Mfn1 in abnormalities of mitochondrial dynamics during the development of cardiomyocyte hypertrophy. To examine this, miR-153-3p antagomir was transfected into cardiomyocytes with or without Mfn1-siRNA, and then cells were exposed to ISO. Treatment with miR-153-3p antagomir could reverse the ISO-induced suppression of Mfn1 protein expression, but this effect was attenuated in cells co-transfected with Mfn1 siRNA and miR-153-3p antagomir (Figure 6A). Also, ISO-induced increase in the percentage of mitochondrial fragmentation and cell surface area was remarkably reduced in cells treated with antagomir alone, and this effect was blocked upon silencing of Mfn1 (Figure 6B-E). These results indicated that miR-153-3p directly targets Mfn1 and regulates its expression to trigger mitochondrial fission during the development of pathological hypertrophy.

### NFATc3 activates miR-153-3p transcription and promotes mitochondrial fission in hypertrophied cardiomyocytes

**Figure 7:**
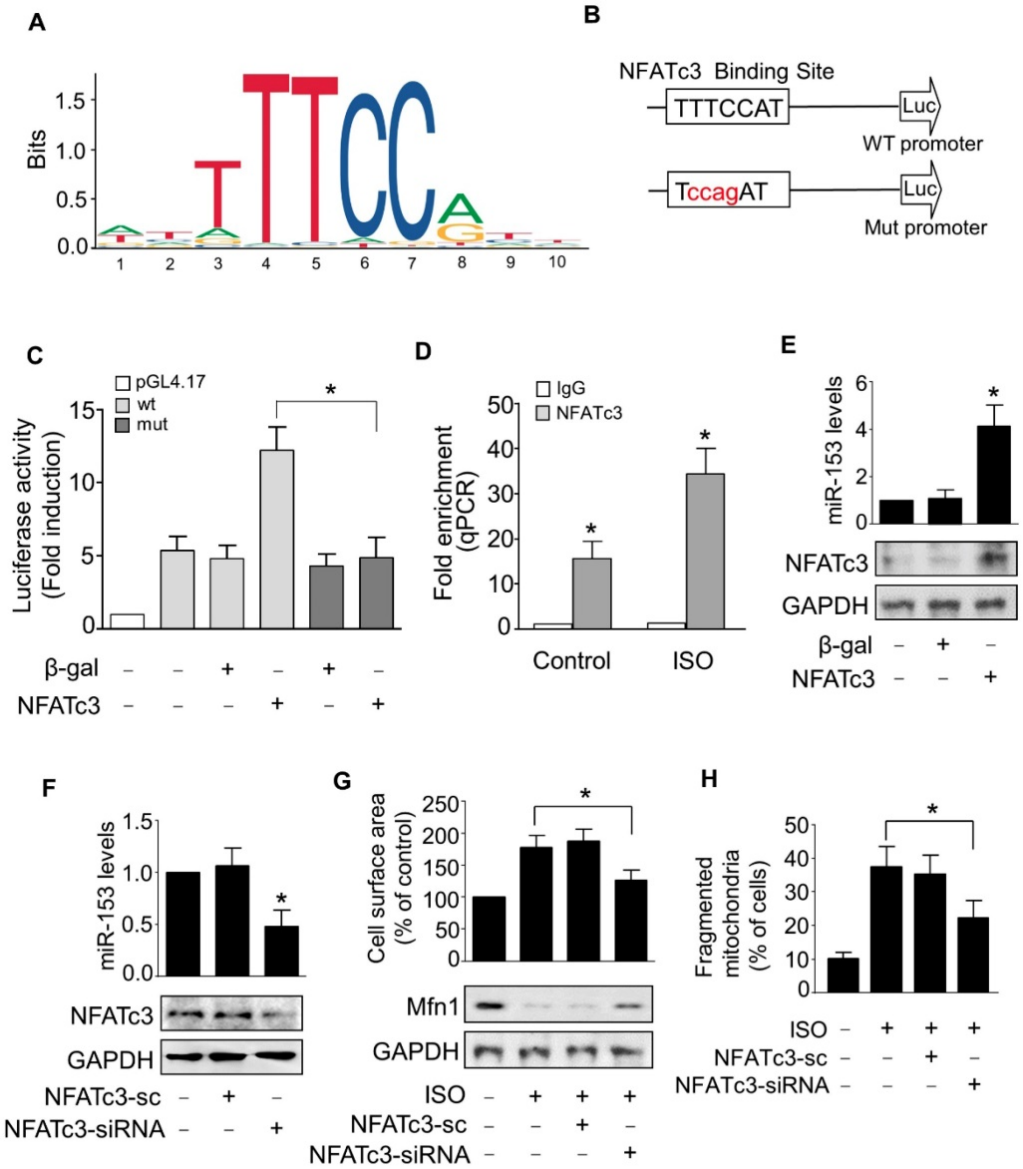
** miR-153-3p is a direct transcriptional target of NFATc3. (A** and** B)** Images showing the potential NFATc3 binding site in the mouse miR-153-3p promoter region.** (C)** NFATc3 binds to the miR-153-3p promoter. Cardiomyocytes were treated with adenoviral β-gal or NFATc3 along with the luciferase constructs of empty vector (pGL-4.17), wild-type miR-153-3p promoter (wt) or the promoter with mutations in NFATc3 binding site (mut) and luciferase activity was measured. **(D)** CHIP-qPCR analysis of NFATc3 binding to the miR-153-3p promoter. **(E)** Overexpression of NFATc3 increases miR-153-3p level. Cardiomyocytes were infected with adenovirus harboring NFATc3 or β-gal and then the level of miR-153-3p was measured by qRT-PCR (upper panel) and NFATc3 level detected by immunoblot (lower panel). **(F)** Knockdown of NFATc3 reduces miR-153-3p level. Cardiomyocytes were infected with adenovirus carrying NFATc3-siRNA or NFATc3-sc and the level of miR-153-3p was determined by qRT-PCR (upper panel) and NFATc3 level detected by immunoblot (lower panel).** (G** and **H)** Knockdown of NFATc3 inhibits ISO-induced increase of the cell surface area and mitochondrial fission. Cardiomyocytes were infected with adenovirus harboring NFATc3-siRNA or NFATc3-sc and then treated with ISO. The cell surface area (G, upper panel), Mfn1 protein level (G, lower panel) and mitochondrial fission (H) were determined. *P < 0.05.

Next, we examined the upstream activator of miR-153-3p expression in pathological hypertrophy. The expression levels of miRNAs can be regulated at the transcriptional level by many transcription factors 35, 36. Therefore, we analyzed the promoter region of mouse miR-153-3p and found that it contained a potential binding site (TTTCCAT) for NFATc3 located from -4402 to -4396 (Figure 7A-B). To validate the binding of NFATc3 to the miR-153-3p promoter, we performed luciferase assay in cells co-transfected with NFATc3 and pGL4.17 vector carrying the wild type or mutant form of miR-153-3p promoters. The luciferase activity was increased in wild-type (WT) miR-153-3p promoter-transfected cells, while mutations in the NFATc3 binding site abolished the luciferase activity (Figure 7C). This result indicated that NFATc3 bound to the promoter region of miR-153-3p to activate its transcription. The ChIP-qPCR analysis showed that NFATc3 bound to the miR-153-3p promoter, and ISO treatment further enhanced the binding of NFATc3 to the miR-153-3p promoter (Figure 7D). Also, overexpression of NFATc3 significantly increased the expression level of miR-153-3p (Figure 7E). In contrast, knockdown of NFATc3 markedly decreased the expression of miR-153-3p mRNA (Figure 7F). Furthermore, knockdown of NFATc3 attenuated ISO-induced hypertrophic growth of cardiomyocytes (Figure 7G) and mitochondrial fission (Figure 7H) in cardiomyocytes. Taken together, these data suggested that miR-153-3p is a potential transcriptional target for NFATc3, which activates its expression at the transcriptional level mediating mitochondrial fission and hypertrophy in cardiomyocytes.

## Discussion

Mitochondrial dynamics is a tightly controlled process that maintains the shape and functions of mitochondria. The balancing of mitochondrial dynamics is not only essential for maintaining normal cellular metabolism but also required for the proper functioning of organs with high-energy demand such as the heart. The imbalance of mitochondrial dynamics leads to mitochondrial dysfunction and contributes to the development of cardiac hypertrophy and heart dysfunction 11, 12, 37, 38.

In this study, we provide evidences that miR-153-3p acts as a pro-hypertrophic factor by regulating mitochondrial dynamics in cardiomyocytes. We found that miR-153-3p was up-regulated in response to stimulation of hypertrophy, and increased expression of miR-153-3p accelerated mitochondrial fission and hypertrophic response. Also, translation of Mfn1 was down-regulated by miR-153-3p, and its decrease during cardiomyocyte hypertrophy led to increased mitochondrial fission events and altered mitochondrial dynamics. Our study further revealed that NFATc3 upregulated miR-153-3p expression by directly binding to its promoter in hypertrophied cardiomyocytes. Interestingly, therapeutic inhibition miR-153-3p or NFATc3 significantly reversed the expression level of Mfn1 protein and effectively reduced the hypertrophic response in cardiomyocytes as well as cardiac dysfunction in hypertrophied hearts. Thus, we have shown that NFATc3, miR-153-3p, and Mfn1 constitute a novel axis to regulate mitochondrial dynamics during cardiomyocyte hypertrophy.

The mitochondrial dysfunction due to changes in mitochondrial dynamics is critically involved in the development of cardiac hypertrophy. Many endogenous factors alter mitochondrial dynamics in cardiac myocytes during the pathological hypertrophy. The hypertrophy stimulants such as leptin play a central role in the pathophysiology of many cardiovascular problems and can cause metabolic remodeling and cardiac contractile dysfunction 39, 40. A recent study found that leptin acts as a pro-hypertrophic factor by promoting mitochondrial fission process and mitochondrial dysfunction through calcineurin-pathway mediated dephosphorylation of Drp1 and its translocation to mitochondria 11. The dysregulation of fission or fusion processes due to the enhanced activity of fission proteins such as Drp1 38 or suppressed expression/activity of fusion proteins such as Mfn1 37 triggers aberrant mitochondrial fission and promotes hypertrophic response. Consistent with the reports mentioned above, our results also demonstrated that a hypertrophic stimulant, ISO, induced mitochondrial fission by suppressing Mfn1 protein expression, which led to hypertrophy in cardiomyocytes. We further identified that Mfn1 could block ISO-induced mitochondrial fission and hypertrophy in cardiomyocytes. We have also shown that aberrant mitochondrial dynamics contributed to cardiac hypertrophy, and, therefore, proteins associated with this phenomenon could be potential targets for the treatment and intervention of cardiac hypertrophy.

Numerous studies reported that miRNAs regulate mitochondrial fission in various cell types 41, 42. However, few studies have focused on miRNA-mediated regulation of mitochondrial network dynamics during cardiac hypertrophy. For instance, miR-324-5p blocked mitochondrial fission through suppression of Mtfr1 translation 26. On the other hand, miR-421 promoted mitochondrial fragmentation in cardiomyocytes by inhibiting Pink1 translation 42. The molecular functions of miR-153-3p are well established in various types of cancer. In glioma cells, miR-153-3p enhanced the radiosensitivity by targeting BCL2 43. In melanoma cells, miR-153-3p targeted SNAI1 to inhibit cell proliferation and invasion 44. It could also regulate the proliferation and invasion of breast cancer cells by targeting KLF15 45. However, to date, there is no report available to show the functional effects of miR-153-3p on cardiac diseases. We, for the first time, demonstrated that miR-153-3p mediated hypertrophy of cardiomyocytes both *in vitro* and *in vivo*. We also found that the knockdown of miR-153-3p attenuated mitochondrial fission and the progression of hypertrophy in cardiomyocytes and hypertrophied mice hearts. Furthermore, we showed that miR-153-3p executed its effects by inhibiting Mfn1 translation. Therefore, miR-153-3p could be a potential target for the prevention and treatment of pathological cardiac hypertrophy.

NFATc3 is one of the central effectors of signaling pathways in cardiac hypertrophy. The dephosphorylated form of NFATc3 binds to GATA4 in the nucleus and directly activates the expression of cardiac hypertrophy-associated genes 46. Also, NFATc3 upregulates the expression of myocardin, which is a transcriptional cofactor involved in the expression of hypertrophic genes 47. In cardiomyocytes, NFATc3 was reported to trigger mitochondrial fission and apoptotic cell death 26. In this study, we uncovered the link between the increased NFATc3 activity and aberrant mitochondrial fission in cardiac hypertrophy. Silencing of NFATc3 inhibited mitochondrial fission and hypertrophic changes in cardiomyocytes. Mechanistically, NFATc3 activated miR-153-3p transcription and increased its expression, resulting in the post-transcriptional suppression of Mfn1 and reduction at the protein level under hypertrophic conditions. Given that NFATc3 activates many downstream genes associated with cardiac hypertrophy, it would be worth investigating whether NFATc3/miR-153-3p axis is involved in the regulation of other proteins related to mitochondrial dynamics during the development of cardiac hypertrophy.

In recent years, great strides have been made in delineating the molecular mechanisms associated with the development and progression of cardiac hypertrophy and developing efficient diagnostics and effective therapeutics for its treatment 48, 49. In this context, our study has identified a novel signaling pathway consisting of NFATc3, miR-153-3p, and Mfn1 that instigated mitochondrial dynamic imbalance by triggering abnormal mitochondrial fission in cardiomyocyte hypertrophy. Furthermore, inhibition of NFATc3/miR-153-3p or replenishment of Mfn1 (by enforced expression) significantly attenuated hypertrophic response in cardiomyocyte. Thus, our study has identified potential targets for the treatment of cardiac hypertrophy and dysfunction.

## Supplementary Material

Supplementary figures.
