# Supplementary material for: NFATc3-dependent expression of miR-153-3p promotes mitochondrial fragmentation in cardiac hypertrophy by impairing mitofusin-1 expression
Source: Theranostics. 2020 Jan 1;10(2):553–66. doi: 10.7150/thno.37181 (PMC6929994; doi:10.7150/thno.37181)

Supplementary figure legends

Supplementary Figure 1. The effect of miR-153-3p on the regulation of mitochondrial dynamics related proteins. (**A**) Cardiomyocytes were transfected with anta-153-3p or anta-NC, and then were treated with ISO. 24h after treatment, cells were harvested and the expression of Drp1, Fis1, MFF, MAPL and MTP18 were detected by immunoblot. (**B**) Cardiomyocytes were treated as described in (A), Mfn2, OPA1, Parkin and Pink1 levels were detected by immunoblot.

Supplementary Figure 2. The detection of Mfn1 expression levels. (**A**) Cardiomyocytes were infected with wild-type Mfn1-3'UTR adenovirus (Mfn1-3'UTR-wt) and then were transfected with mimic-153-3p or mimic-NC. 24h after transfection, cells were harvested, and Mfn1 expression was detected by immunoblot. (**B**) Cardiomyocytes were infected with adenovirus expressing mutated Mfn1-3'UTR (Mfn1-3'UTR-mut) and then were transfected with mimic-153-3p or mimic-NC. 24h after transfection, cells were harvested, and Mfn1 expression was detected by immunoblot.

Supplementary Figure 1

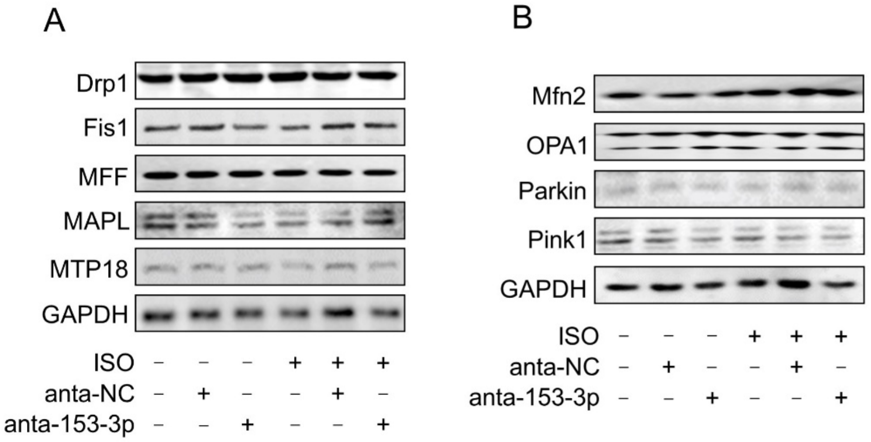

Supplementary Figure 2

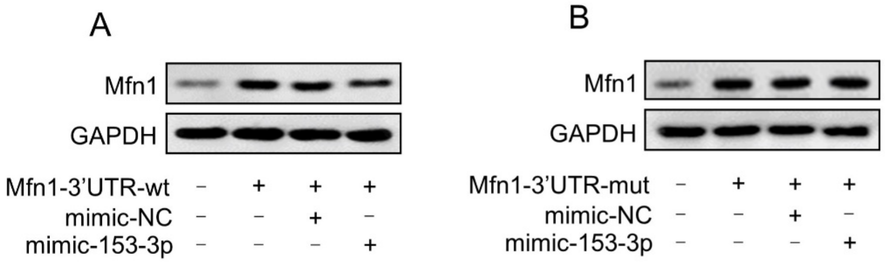

Supplement: Supplementary file 1 — Supplementary figures. [file thnov10p0553s1.pdf]
